# Myofibroblast Expression in Skin Wounds Is Enhanced by Collagen III Suppression

**DOI:** 10.1155/2015/958695

**Published:** 2015-02-19

**Authors:** Mohammed M. Al-Qattan, Mervat M. Abd-Elwahed, Khalid Hawary, Maha M. Arafah, Medhat K. Shier

**Affiliations:** ^1^Department of Surgery, King Saud University, P.O. Box 18097, Riyadh 11415, Saudi Arabia; ^2^College of Medicine Research Center, King Saud University, P.O. Box 18097, Riyadh 11415, Saudi Arabia; ^3^Department of Pathology, King Saud University, Riyadh 11415, Saudi Arabia

## Abstract

Generally speaking, the excessive expression of myofibroblasts is associated with excessive collagen production. One exception is seen in patients and animal models of Ehlers-Danlos syndrome type IV in which the *COL3A1* gene mutation results in reduced collagen III but with concurrent increased myofibroblast expression. This paradox has not been examined with the use of external drugs/modalities to prevent hypertrophic scars. In this paper, we injected the rabbit ear wound model of hypertrophic scarring with two doses of a protein called nAG, which is known to reduce collagen expression and to suppress hypertrophic scarring in that animal model. The higher nAG dose was associated with significantly less collagen III expression and concurrent higher degree of myofibroblast expression. We concluded that collagen III content of the extracellular matrix may have a direct or an indirect effect on myofibroblast differentiation. However, further research is required to investigate the pathogenesis of this paradoxical phenomenon.

## 1. Introduction

In unwounded skin, fibroblasts are in a quiescent state within the extracellular matrix (ECM). Acute wounding results in changes in the microenvironment of the ECM (including the release of cytokines and growth factors) leading to the transformation of fibroblasts into myofibroblasts [1]. Myofibroblasts will have multiple functions such as collagen deposition and mediation of the cross talk within the ECM [2]. In primary wound healing, myofibroblasts undergo apoptosis after 2 weeks [1, 3]. In wounds healing by secondary intention, myofibroblasts persist in the granulation tissue producing collagen and mediating wound contraction [3]. In pathological scarring such as scar hypertrophy and fibrotic conditions (such as scleroderma, pulmonary fibrosis, and Dupuytren's contracture), there is excessive expression of myofibroblasts and excessive production of collagen [1–3]. In other words, the excessive expression of myofibroblasts is almost always associated with excessive collagen deposition, and this is not surprising since myofibroblasts make and deposit collagen in the ECM. Ehlers-Danlos syndrome type IV (EDS IV, also known as the vascular type) is one exception. Patients with EDS IV have a mutation in the* COL3A1* gene resulting in reduced amount of collagen III in their skin and other connective tissue such as the walls of the arteries and intestine. This results in areas of atrophic skin, easy bruising, aneurysms, and spontaneous visceral rupture [4, 5]. However, patients with EDS IV are also known to develop postoperative scar hypertrophy [5] and spontaneous multiple skin keloid-like lesions [4]. Biopsies of these keloidal lesions show abnormal collagen (highly eosinophilic thick collagen bundles lying irregularly) with normal epidermis [4]. In contrast, biopsies of atrophic skin areas show epidermal atrophy, reduced collagen, loss of rote pegs, extravasation of red blood cells, and hemosiderin deposition in the dermis [4]. Mice models of EDS IV are heterozygous mutants known as the “Col III^+/−^ mice” (since EDS IV in humans is autosomal dominant with heterozygous mutations). These mice have similar features to humans including reduced production of collagen III. Volk et al. [6] produced excisional wounds in Col III^+/−^ mice and compared them to similar wounds in normal (Col III^+/+^) mice. Mutant mice had less collagen III but much more myofibroblast expression leading to accelerated wound contraction [6]. The authors could not explain this paradox and thought that further research is needed to investigate if other causes of collagen III deficiency will promote myofibroblast differentiation.

nAG is a salamander-derived protein mediating limb regeneration [7]. In a previous work in our lab, a new nAG gene (suitable for mammals) was designed and cloned. The cloned vector was transfected into primary human fibroblasts resulting in suppression of collagen expression [8]. In another communication we showed that daily local injections of recombinant nAG into the rabbit ear hypertrophic scar model results in lower scar elevation index (SEI) and lower collagen content [9]. Myofibroblast expression was not examined in our previous studies.

In the current paper, we injected rabbit ear wounds with two different doses of recombinant nAG and show that the degree of expression of myofibroblasts is inversely proportional to the degree of collagen III expression.

## 2. Materials and Methods

The experiment was approved by the Ethics Committee of the Medical College of our university.

### 2.1. Preparation of Recombinant nAG and the Rabbit Ear Model

Details of synthesis and preparation of mammalian recombinant nAG protein was described in our previous work [8, 9]. Mammalian recombinant nAG protein was synthesized by Genscript Company (Piscataway, NJ, USA) by using mammalian transient protein expression system (HEK 293-6E cells, mammalian expression rector pTT5). Individual doses (100 nM and 150 nM) were prepared and stored at −80°C.

The hypertrophic scar model in the rabbit ear is a well-established model in the literature [10, 11]. A 7 mm punch biopsy is used to create a wound in the ear removing the epidermis, dermis, and perichondrium. The underlying cartilage acts as a splint to the wound edges and the wound epithelializes in 14 days. An established hypertrophic scar is known to appear at day 28. In studies (including our study) that aim to test the effect of a drug or a modality to decrease the formation of hypertrophic scars (i.e., prevention of scarring), the treatment is commenced at day 14 and wound scarring is assessed at day 28.

### 2.2. Surgical Procedure

Eight white New Zealand rabbits weighing 1.5–2 kg were used. The animals were anaesthetized with intramuscular ketamine (40 mg/kg) and xylazine (4 mg/kg). One wound was created in each ear (one experimental and one control wound). A 7 mm punch biopsy was used to create the wound as described above. Wounds were dressed with normal saline-soaked gauze.

Wound injections were done daily starting at day 14 and ending at day 27. All control wounds (*n* = 8) were injected with 100 *μ*L of saline. Four experimental wounds were injected with 100 nM of recombinant nAG (in 100 *μ*L of saline) and the other four experimental wounds were injected with 150 nM of recombinant nAG (in 100 *μ*L of saline).

### 2.3. Assessment

Assessment was done in experimental and control wounds at day 28 after wounding. Three parameters were assessed: scar elevation index, histology, and quantitative determination of mRNA of collagen I and collagen III.

#### 2.3.1. The Scar Elevation Index (SEI)

SEI was previously described [12] and it represents the ratio of the scar tissue height to the normal tissue below the hypertrophic scar. A ratio of 1 indicates no difference in the wound area height compared with unwounded skin.

#### 2.3.2. Histological Analysis and Masson's Trichrome Staining

Histological sections were fixed with 4% paraformaldehyde, embedded in paraffin, cut in 5 *μ*m sections, and stained with hematoxylin and eosin (H&E). Masson's trichrome staining was done to stain collagen fibers.

#### 2.3.3. Immunohistochemistry

Paraffin-embedded blocks of wounded skin specimens were cut into 3 *μ*m thick sections. The sections were mounted on saline coated slides and were incubated for 20 minutes in hot air oven at 60°C. Tissue sections were deparaffinized with EZ Prep (Ventana, Arizona, USA) at 75°C and were heat-pretreated in Cell Conditioning 1 (CC1; Ventana, Arizona, USA) using “standard cell conditioning” for antigen retrieval at 100°C, and then one drop of inhibitor solution was put for four minutes at 37°C and then tissue sections were washed. The slides were incubated 30 minutes at 37°C with diluted 1 : 200 anti-*α*-smooth muscle actin (*α*-SMA) or TGF*β*1 monoclonal antibody (Abcam, UK). Then ultraview universal HRP multimer as secondary antibody was added. The immunostained proteins were visualized using a copper-enhanced DAB reaction. Immunostained sections were reviewed by Olympus BX51 light Microscope and DP72 Olympus Digital Camera (magnification 200x and 400x) (Olympus America Inc., Center Valley, PA, USA).

#### 2.3.4. Real Time PCR for Collagen mRNA Quantitation

RNA was extracted from tissue samples using RNeasy Protect Mini Kit (Qiagen, Valencia, CA) and quantified by nanodrop. 100 ng of total RNA was reverse-transcribed by using Rotor-Gene Multiplex PCR Kit (Qiagen, Valencia, CA). Relative mRNA levels were calculated by using the ΔΔCt method and GAPDH as a reference gene. Primers and probes were mentioned in our previous published work [9]. Experiments were done three times at least.

### 2.4. Statistical Analysis

The SEI and RNA extraction data were expressed as mean ± SD. The differences between the values of the two experimental groups (the 100 nM nAG group and the 150 nM nAG group) as well as the control group (the saline without nAG group) were analyzed using the independent sample *t*-test (for SEI) and the Mann-Whitney test (for RNA extraction data). *P* < 0.05 indicated a significant difference.

## 3. Results

**Figure 1:**
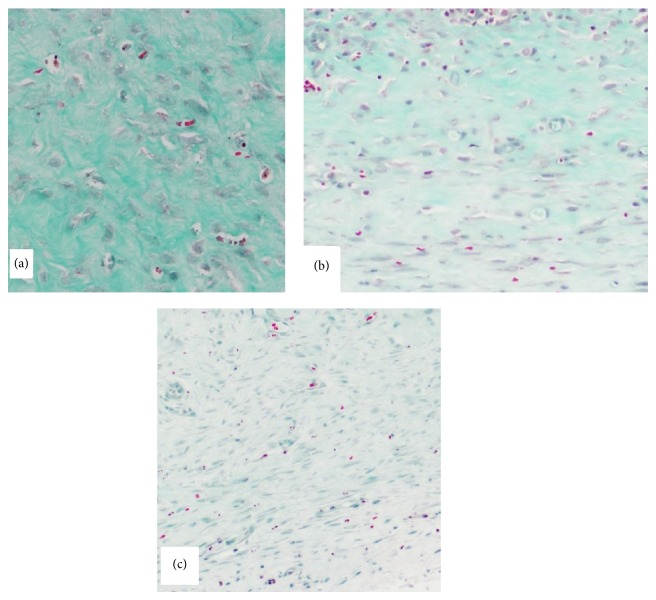
Masson's trichrome staining of the wound in the three groups. (a) A control wound showing thick, dense, and disorganized collagen fibers (magnification 40x). (b) An experimental 100 nM nAG wound showing thin more regularly arranged collagen fibers with increased number of small blood vessels (magnification 40x). (c) An experimental 150 nM nAG wound showing faint staining with sparse thin collagen bundles and increased vascularity (magnification 40x).

**Figure 2:**
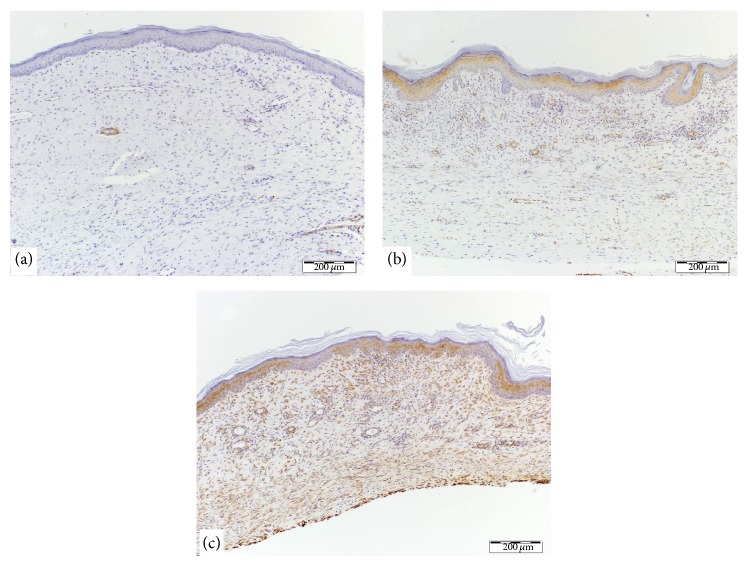
*α*-smooth muscle actin stain (brown color) in the three groups. (a) A control wound showing minimal staining which is mainly confined to the few dermal blood vessels (magnification 10x). (b) An experimental 100 nM nAG wound showing moderate staining which is more pronounced in the upper dermis (magnification 10x). (c) An experimental 150 nM nAG wound showing intense staining of the entire dermis (magnification 10x).

At 28 days, the SEI of the control, the 100 nM nAG, and the 150 nM nAG groups were 1.37 ± 0.08, 1.19 ± 0.05, and 1.07 ± 0.07, respectively. The differences between the three groups were significant (*P* < 0.005). Masson's trichrome staining showed thick, dense, and disorganized collagen fibers in the control wounds (Figure 1(a)); thin more regularly arranged fibers with increased number of small blood vessels in the 100 nM nAG experimental group (Figure 1(b)); and faint staining with sparse thin collagen bundles and increased vascularity in the 150 nM nAG experimental group (Figure 1(c)). The vascularity in the 100 nM nAG groups was more pronounced in the upper dermis, whereas it involved the entire dermis in the 150 nM nAG group. *α*-SMA stains both blood vessels and myofibroblasts. At 28 days after wounding, the control wound had minimal positive *α*-SMA staining confined to the few dermal blood vessels (Figure 2(a)). The experimental 100 nM nAG group had moderate positive *α*-SMA staining which was more pronounced in the upper dermis (Figure 2(b)). Finally, the experimental 150 nM nAG group had intensely positive *α*-SMA staining of the entire dermis (Figure 2(c)).

**Figure 3:**
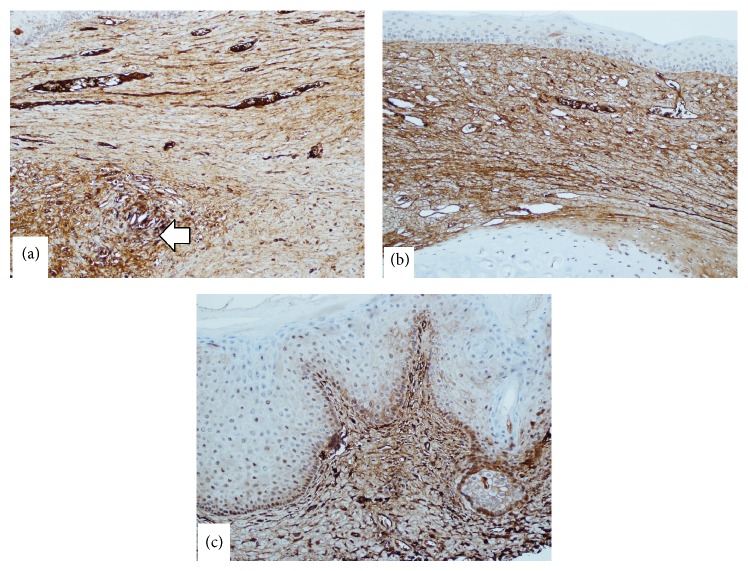
TGF*β*1 immunohistochemical staining. (a) A control wound showing relatively mild heterogeneous positivity, although one focal area is more positive (arrow) (magnification 20x). (b) An experimental 100 nM nAG wound showing diffuse moderate staining indicating moderate positivity (magnification 20x). (c) An experimental 150 nM nAG wound showing intense staining (magnification 20x).

TGF*β*1 immune staining showed relatively mild heterogeneous positivity in control wounds (Figure 3(a)), diffuse moderate positivity in the 100 nM nAG group wounds (Figure 3(b)), and intense staining in the 150 nM nAG group wounds (Figure 3(c)).

Compared to controls, the relative mRNA expression of collagen I and collagen III in both experimental groups is shown in Table 1. Both experimental groups had significantly (*P* < 0.05) less collagen I and collagen III contents than controls. When the two experimental groups were compared to each other, there was no significant difference (*P* > 0.05) in collagen I content, but the high dose nAG group had significantly more collagen III suppression (*P* < 0.05) than the low dose nAG group.

## 4. Discussion

Our study showed the persistent high expression of myofibroblasts in wounds deficient in collagen. Both experimental groups had similar reductions of collagen I content. However, collagen III expression was most significantly reduced in the 150 nM nAG group and this corresponded with the highest degree of myofibroblast expression. This indicates that it is the suppression of collagen III (and not collagen I) which induced the increased expression of myofibroblasts. Further research is required to investigate if this paradoxical phenomenon (low collagen III content with high myofibroblast expression) will also be seen with other modalities used to prevent hypertrophic scars.

The creation of a wound with very low collagen content and a high degree of myofibroblast expression is interesting from the scientific point of view. Myofibroblasts are known to have mature focal adhesions in their cell membrane [1–3]. These focal adhesions are complexes of many molecules including integrins and kinases. Myofibroblasts will not only produce collagen but can also “sense” tension in the ECM through these focal adhesions which act like receptors [2]. The most two important receptors mediating tension “sensing” and myofibroblast activity are the *α*Ѵ*β*3 and *α*5*β*1 integrin receptors. Vitronectin binds mainly to *α*Ѵ*β*3 receptors, whereas fibronectin binds mainly to *α*5*β*1 receptors [1–3]. Zoppi et al. [13] have shown that fibroblasts and myofibroblasts with* COL3A1* gene mutations will not only produce less collagen III but will also recruit the *α*Ѵ*β*3 instead of *α*5*β*1 integrin receptors. Hence, it is possible that the low collagen III expression in the ECM will have an effect on myofibroblasts through changes in the “tension” sensing receptors.

It is well known that TGF*β*1 is the main stimulant of myofibroblast differentiation and stimulation of excessive collagen synthesis [14]. Our current study demonstrated the paradox of high myofibroblast expression in the presence of low collagen content. Hence, it seemed reasonable to study the expression of TGF*β*1 within this paradoxical environment. Our study demonstrated that the intensity of TGF*β*1 expression correlates with the intensity of myofibroblast expression. In our previous study [8] we showed that the suppressive effect of nAG on collagen synthesis is evident even with TGF*β*1 stimulation. One possible explanation of the sequence of events in our model would be the primary suppression of collagen III by nAG. This may have caused a biofeedback mechanism through which TGF*β*1 is stimulated, which will then stimulate myofibroblast differentiation. Since the nAG effect on collagen is dominant over the TGF*β*1 effect on collagen, collagen III remained low despite the high TGF*β*1 expression.

Finally, our findings may have clinical relevance. For example, Dupuytren's cords are now being treated with direct injection of collagenase [15]. Collagenases will breakdown collagen within the cords and this allows the surgeon to break the cord by hyperextending the fingers [15]. Recurrence of cord contracture [16] and extensive deep tissue scarring [17] are well-known complications of this management approach. It is possible that the injection of collagenase will not only reduce the collagen content of the cord but will also induce myofibroblast expression leading to recurrence of contractures or deep-tissue scarring.

## 5. Conclusion

Myofibroblast expression in the rabbit ear model is enhanced by collagen III suppression. The pathogenesis of such a paradox needs further research but is clinically relevant.

## Figures and Tables

**Table 1:** “Relative” collagen I and collagen III contents in experimental groups compared to the control group (the collagen content in the control group is considered as 1).

	Relative collagen I content	Relative collagen III content
Control	1	1
Low dose nAG experimental group	0.16 ± 0.06	0.5 ± 0.11
High dose nAG experimental group	0.15 ± 0.07	0.1 ± 0.02

Both experimental groups had significantly (*P* < 0.05) less collagen I and collagen III contents than controls. When the two experimental groups were compared to each other, there was no significant difference (*P* > 0.05) in collagen I content but the high dose nAG group had significantly more collagen III suppression (*P* < 0.05) than the low dose nAG group.
